# Suppression of Low-Frequency Gamma Oscillations by Activation of 40-Hz Oscillation

**DOI:** 10.1093/cercor/bhab381

**Published:** 2021-10-23

**Authors:** Shunsuke Sugiyama, Tomoya Taniguchi, Tomoaki Kinukawa, Nobuyuki Takeuchi, Kazutaka Ohi, Toshiki Shioiri, Makoto Nishihara, Koji Inui

**Affiliations:** Department of Psychiatry, Gifu University Graduate School of Medicine, Gifu 501-1194, Japan; Department of Anesthesiology, Nagoya University Graduate School of Medicine, Nagoya 466-8560, Japan; Department of Anesthesiology, Nagoya University Graduate School of Medicine, Nagoya 466-8560, Japan; Department of Psychiatry, Aichi Medical University, Nagakute 480-1195, Japan; Department of Psychiatry, Gifu University Graduate School of Medicine, Gifu 501-1194, Japan; Department of Psychiatry, Gifu University Graduate School of Medicine, Gifu 501-1194, Japan; Multidisciplinary Pain Center, Aichi Medical University, Nagakute 480-1195, Japan; Department of Functioning and Disability, Institute for Developmental Research, Aichi Developmental Disability Center, Kasugai 480-0304, Japan; Section of Brain Function Information, National Institute for Physiological Sciences, Okazaki 444-8787, Japan

**Keywords:** auditory steady-state response, GABAergic interneuron, magnetoencephalography, N-methyl-d-aspartic acid, oscillation

## Abstract

Gamma oscillations have received considerable attention owing to their association with cognitive function and various neuropsychiatric disorders. However, interactions of gamma oscillations at different frequency bands in humans remain unclear. In the present magnetoencephalographic study, brain oscillations in a wide frequency range were examined using a time-frequency analysis during the 20-, 30-, 40-, and 50-Hz auditory stimuli in 21 healthy subjects. First, dipoles for auditory steady-state response (ASSR) were estimated and interaction among oscillations at 10–60 Hz was examined using the source strength waveforms. Results showed the suppression of ongoing low-gamma oscillations at approximately 30 Hz during stimulation at 40 Hz. Second, multi-dipole analyses suggested that the main dipole for ASSR and dipoles for suppressed low-frequency gamma oscillations were distinct. Third, an all-sensor analysis was performed to clarify the distribution of the 40-Hz ASSR and suppression of low-frequency gamma oscillations. Notably, the area of suppression surrounded the center of the 40-Hz ASSR and showed a trend of extending to the vertex, indicating that different groups of neurons were responsible for these two gamma oscillations and that the 40-Hz oscillation circuit have specific inhibitory innervation to the low-gamma circuit.

## Introduction

Neural oscillation is a rhythmic pattern of neural activity that covers frequencies from approximately 0.05 to 500 Hz in the central nervous system (Buzsáki and Draguhn 2004). Within this spectrum, oscillations at gamma frequencies (20–80 Hz) have been associated with a variety of cognitive processes, including sensory binding (Engel et al. 2001), attentional selection (Fries et al. 2001; Gregoriou et al. 2009), and memory (Lisman 1999; Miller et al. 2018), which has attracted the interest of researchers. In the generation of gamma oscillations, inhibitory interneurons play a pivotal role (Bartos et al. 2007). Studies investigating gamma oscillations have been conducted on various animals and brain regions, including slice preparations (Buzsáki and Wang 2012). In addition to the findings of these studies, theoretical research has further elucidated the generation mechanism (Whittington et al. 2000; Tiesinga and Sejnowski 2009). These studies have proposed two major mechanisms for the generation of gamma oscillation, the interneuronal network gamma mechanism and the pyramidal-interneuronal network gamma mechanism (Whittington et al. 2000; Tiesinga and Sejnowski 2009); in both mechanisms, inhibitory interneurons play an essential role.

Another important aspect of gamma oscillations is that they can be manipulated noninvasively, which enables observation of changes in oscillatory activity during *in vivo* studies. One famous example is the attentional modulation of gamma oscillations (Tiesinga and Sejnowski 2009). The auditory steady-state response (ASSR) has been widely used for experimental observations of humans. ASSR is an electrophysiological response driven by a train of auditory stimuli delivered at a sufficiently high rate. ASSR reaches the maximum amplitude at approximately 40 Hz and subsequently decreases at higher rates (Galambos et al. 1981; Ross et al. 2000; Pastor et al. 2002; Picton et al. 2003). The simplest theory of the generation mechanism of ASSR is that it is composed of a superposition of auditory middle latency response components (Plourde et al. 1991; Galambos and Makeig 1992; Bohórquez and Ozdamar 2008); however, the theory does not explain the apparent frequency selectivity of ASSR. Several findings dispute this theory and propose that ASSR instead represents intrinsic oscillatory processes in auditory pathways. For example, in magnetoencephalography (MEG) studies, the 40-Hz ASSR evolves temporally 200 ms after the onset of a stimulus (Ross et al. 2002), while phase synchronization continues after the offset of a stimulus (Santarelli and Conti 1999). In addition, the 40-Hz ASSR is disrupted by a salient sensory stimulus (Sugiyama et al. 2021), which persists longer than the offset of a stimulus (Ross et al. 2005b). Because a prolonged disruption of the 40-Hz ASSR cannot be simulated by the superposition of middle latency response components, it was suggested that a perturbing stimulus resets the oscillations and shifts back the ASSR phase to that of the driving source (Ross et al. 2005b). Therefore, the 40-Hz ASSR is considered to reflect endogenous gamma oscillations.

In the present study, we focused on the modulation of ongoing brain gamma oscillations based on the 40-Hz ASSR as a biomarker. Cross-frequency coupling is the phenomenon in which one frequency band modulates the activity of a different frequency band (Hyafil et al. 2015). Many studies have shown cross-frequency coupling between slower oscillations in theta (4–8 Hz) or alpha (8–12 Hz) frequency bands and gamma oscillations in animals (Tort et al. 2008; Belluscio et al. 2012; Scheffer-Teixeira et al. 2012) and humans (Canolty et al. 2006; Axmacher et al. 2010; Roux et al. 2013). To illustrate, an increase in alpha oscillations results in decreased gamma activity in the human visual area (Jensen et al. 2014). However, to the best of our knowledge, the interactions between gamma oscillations at different frequency bands in humans have not been investigated. We recorded the MEG signals emitted under repetitive auditory stimuli at 20, 30, 40, and 50 Hz and performed time-frequency analysis to investigate changes in the oscillation amplitude in a wide frequency range. This simple paradigm was used to investigate the interactions between the circuits that are responsible for oscillations at different frequencies. MEG has high spatial resolution, which is favorable for dipole estimation. MEG signals recorded using planar-type gradiometers were sufficiently powerful to detect the largest signal over local cerebral sources; this was consequently beneficial for the present study to elucidate the scalp distribution of brain oscillations. In a rat slice preparation, Middleton et al. (2008) revealed that interactions between two oscillation circuits of different frequencies contributed to the oscillatory pattern in gamma bands, particularly at around 30 and 40 Hz. We considered that similar interactions between gamma oscillations at different frequencies might exist in humans, which could facilitate further clarification of the mechanisms of gamma oscillations and the pathophysiology of certain neuropsychiatric disorders including Alzheimer’s disease (Palop et al. 2007; Verret et al. 2012) and schizophrenia (Spencer et al. 2003; Lewis et al. 2005) that are associated with disrupted gamma oscillations.

## Materials and Methods

### Ethics Statement

This study was approved by the Ethics Committee of the National Institute for Physiological Sciences, Okazaki, Japan, and was conducted in accordance with the Declaration of Helsinki. Written informed consent was obtained from all participants.

### Subjects

We enrolled 21 healthy volunteers (5 women and 16 men) aged 25–58 (mean, 33.4) years. The participants had no history of mental or neurological disorders or substance abuse in the last 2 years and were not taking any medication at the time of testing. The patients had a hearing threshold of <25 dB at 1000 Hz, which was assessed using an audiometer (AA-71, Rion, Tokyo, Japan).

### Auditory Stimulation

Auditory stimuli were induced using repeats of a brief pure tone. The pure tone was 800 Hz in frequency, and the sound pressure level was 70 dB. There were four pure tones played in durations of 50, 33.3, 25, and 20 ms (rise/fall, 5 ms), with auditory stimuli were composed of trains of 14, 21, 28, and 35 pure tones, respectively. Therefore, there were four frequency conditions of 20-, 30-, 40-, and 50-Hz with a total duration of 700 ms. The sound stimulus was presented binaurally via earpieces (E-A-RTONE 3A, Aero Company, Indianapolis, IN, United States of America), and the sound pressure was controlled with an audiometer (AA-71, RION, Tokyo, Japan).

### MEG Recordings

Magnetic signals were recorded using a 306-channel whole-head MEG system (Vector-view, Elekta Neuromag, Helsinki, Finland) composed of 102 identical triple sensor elements. Each sensor element included two orthogonal planar gradiometers and one magnetometer coupled with a multi-superconducting quantum interference device, which provided three independent measurements of magnetic fields. The MEG signals were recorded using 204 planar-type gradiometers. Prior to recording, a current was fed to four head position indicator (HPI) coils placed strategically at sites that would obtain the precise location of the head with respect to the sensors, and the resulting magnetic fields were measured using a magnetometer; this approach enabled alignment of the individual head coordinate system with the magnetometer coordinate system. A 3D digitizer was used to measure the four HPI coils attached on the individual’s head with respect to the three anatomical landmarks. The X-axis was fixed with preauricular points, with the right being the positive direction. The positive Y-axis passed through the nasion, while the Z-axis pointed upward. Signals were recorded using a band-pass filter of 0.1–300 Hz and were digitized at 1000 Hz. Epochs with MEG signals of >2.7 pT/cm were excluded from the average.

The experiments were performed in a quiet, magnetically shielded room. The participants were instructed to sit in a chair and watch a silent movie projected on a screen 1.5 m in front of them and to ignore the auditory stimulation. The four auditory stimuli were randomly presented with an even probability using a stimulus onset asynchrony of 1250 ms; i.e., an inter stimulus interval of 550 ms. A total of at least 100 artifact-free epochs were averaged for each stimulus per participant.

### Time-Frequency Analysis

Time-frequency analyses were applied to MEG signals at 500 ms before and 1000 ms after the onset of auditory stimulation. Three kinds of data were used: source strength waveforms of a two-dipole model, those of a multi-dipole model, and MEG signals of all sensors. For dipole analyses, the Brain Electrical Source Analysis software package (GmbH, Grafefling, Germany) was used. Time-frequency analyses were performed for each epoch to calculate the amplitude and inter-trial coherence of evoked oscillations for frequencies from 10 to 60 Hz with 2 Hz frequency resolution using Morlet wavelet transformation every 25 ms. Results of the analysis were then averaged across all epochs.

#### The Two-Dipole Model

**Figure 1:**
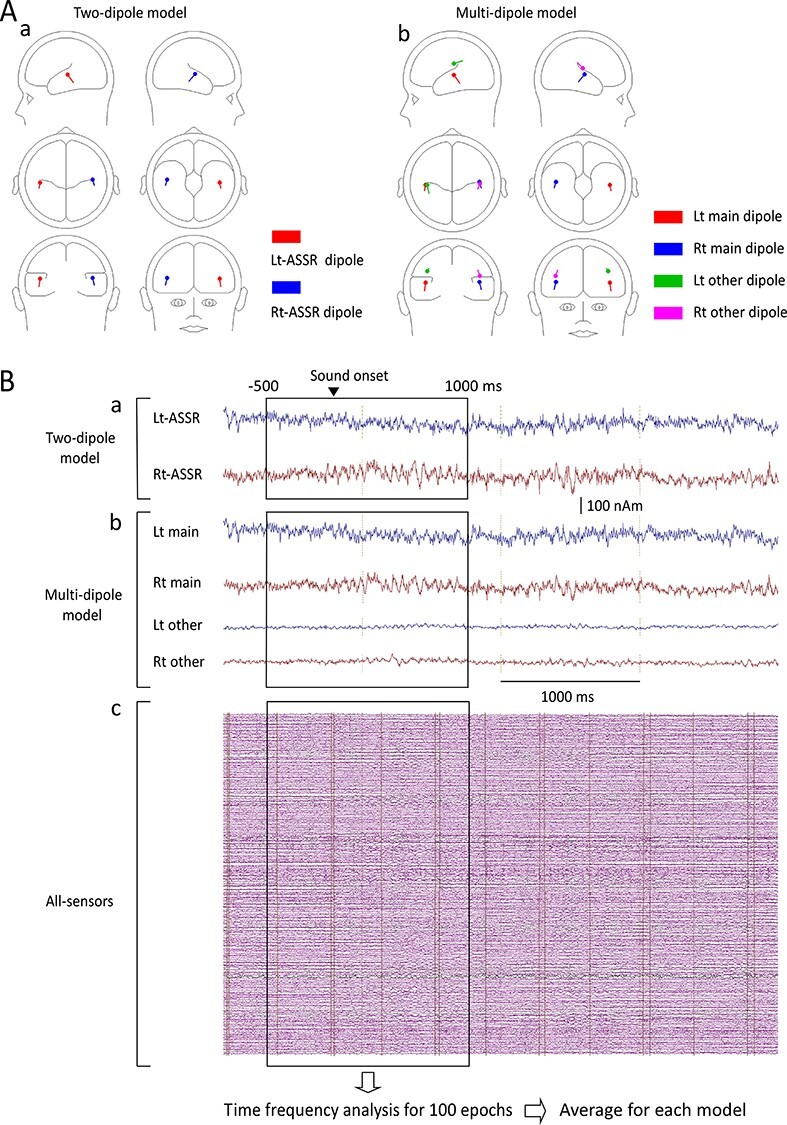
**Three methods for time-frequency analysis.** Dipole locations and orientations in the two-dipole (Aa) and multi-dipole (Ab) models. To perform time-frequency analyses, source strength waveforms were used for the two-dipole (Ba) and multi-dipole (Bb) models, while magnetic signals recorded from all 204 gradiometers were used for the all-sensor analysis (Bc).

First, we simply performed a two-dipole analysis that estimated an equivalent current dipole for the main component of ASSR by hemisphere. The MEG waveforms of the 20-, 30-, 40-, and 50-Hz conditions were averaged across 100 trials and were filtered with band-pass filters of 17.5–22.5, 27.5–32.5, 37.5–42.5, and 47.5–52.5 Hz, respectively, to selectively extract that cortical activity occurring during the oscillation at the stimulus frequency. In each condition, the equivalent current dipole for the main component of ASSR was estimated per hemisphere in a time window of 200–700 ms. The left and right dipoles for the main components of 40-Hz ASSR for all 21 subjects were estimated. However, we could not estimate the dipoles under the 20-, 30-, and 50-Hz conditions for 22, 16, and 6 hemispheres, respectively. Therefore, the obtained two-dipole model of the 40-Hz condition was applied to the MEG signals under all conditions, and time-frequency analysis was performed on the source strength waveforms obtained by the two-dipole model. Dipoles were estimated to be located in the transverse gyrus (Fig. 1Aa). By applying the two-dipole model to MEG signals, source strength waveforms were obtained for the time-frequency analysis (Fig. 1Ba). For statistical analyses, the average amplitude of the baseline from 500 to 0 ms before the onset of auditory stimulation (Pre) and the average amplitude of 200–700 ms (Post) were compared using two-way repeated-measures analysis of variance (ANOVA) with hemisphere and PrePost as independent variables for each frequency under all conditions. Post hoc paired comparisons were performed using Bonferroni-adjusted *t*-tests when the hemisphere × PrePost interaction effects were significant. All statistical analyses were performed with the level of significance set at 0.05. As for the inter-trial coherence, similar to the amplitude of oscillations, the average coherence of the baseline from 500 to 0 ms before the onset of auditory stimulation and that of 200–700 ms were calculated for each frequency under all conditions.

#### Multi-Dipole Model

As the next step, we attempted to perform a multi-dipole analysis to identify whether we could separate oscillations at different frequencies as different dipoles, following a previous study (Inui et al. 2004). In brief, model adequacy was assessed by examining the percentage of variance and the *F*-ratio. New dipoles with an *F*-ratio of *P* values less than 0.05 were considered significant. The similar dipoles that were estimated under some frequency conditions for both the main dipole and other dipoles were averaged across four frequency conditions. After these procedures were completed, a multi-dipole model was created for each subject. As shown in Figure 1Ab, dipoles other than the main dipole seemed to be located around the main dipole. The source strength waveforms obtained from all estimated dipoles in each subject were used for the analyses (Fig. 1Bb). The discriminant analysis revealed that the location and orientation of the dipoles did not significantly differ between dipoles of the two-dipole model and the main dipoles of the multi-dipole model (*P* > 0.99). There were four subjects with a two-dipole model (Lt and Rt main dipoles), five subjects with a three-dipole model (Lt and Rt main dipoles + Lt other dipole), four subjects with a three-dipole model (Lt and Rt main dipoles + Rt other dipole), and eight subjects with a four-dipole model (Lt and Rt main dipoles + Lt and Rt other dipoles). Similar to the analysis used for the two-dipole model, time-frequency analysis was performed on the source strength waveforms obtained by the multi-dipole model for all conditions and all subjects. Regarding the main dipole, the average amplitude from 500 to 0 ms before the onset of auditory stimulation (Pre) and the average amplitude of 200–700 ms (Post) were compared using two-way ANOVA with hemisphere and PrePost as independent variables for each frequency in all conditions. Post hoc paired comparisons were performed using Bonferroni-adjusted t-tests when the hemisphere × PrePost interaction results were significant. Regarding other dipoles, the average amplitude of 200–700 ms was compared between the baseline and the left hemisphere for 13 subjects and the right hemisphere for 12 subjects using a multiple paired *t*-test instead of ANOVA, from 20 to 38 Hz, because there were only eight subjects who had other dipoles in both hemispheres. All statistical analyses were performed with the significance level set at 0.05.

#### All-Sensor Analysis

Using 204 gradiometers, MEG signals were recorded and subjected to time-frequency analysis for the 40-Hz condition (Fig. 1Bc). To observe the distribution of changes in oscillatory activity, the amplitude of the pair gradiometers was averaged for all 102 sensor locations. Then, the ratios of the amplitude of 200–700 ms to the baseline at 20–32 and 38–42 Hz were calculated for each location. To increase the accuracy of the spatial distribution, the relative location of sensors was aligned across participants using the sensor location with the largest 40-Hz ASSR in each hemisphere. Data for the largest 40-Hz ASSR were averaged across participants. The most common sensor location of the largest response for the right hemisphere, for example, was M1322/1323; therefore, the averaged data were plotted there. There were eight sensor locations that surrounded M1322/1323, so data for the eight locations surrounding the largest 40-Hz ASSR location were averaged across participants and the results were plotted at each of the eight locations around M1322/1323. The eight sensor locations were further surrounded by 16 locations, and the relative location of them was aligned across participants by the same procedures. After completing alignment of all the 102 locations, we obtained a whole head map of the amplitude distribution.

## Results

### Results of Two-Dipole Model Analysis

**Figure 2:**
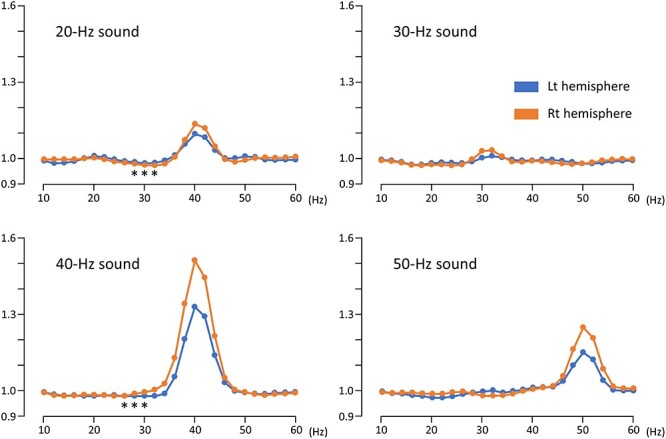
**Changes in the amplitude of oscillations in the two-dipole model.** The vertical axis indicates the ratio of the amplitude relative to the baseline. Note that there are small but significant reductions in the amplitude of oscillations at 28–32 Hz in the 20-Hz sound condition and at 26–30 Hz in the 40-Hz sound condition, as indicated by asterisks (*P* < 0.05).

The results of two-way ANOVA (hemisphere × PrePost) for each frequency band revealed that PrePost significantly affected the amplitude of oscillations around the stimulus frequency; at 30 and 32 Hz in the 30-Hz condition (*P* < 0.032), at 36–46 Hz in the 40-Hz condition (*P* < 0.011), and at 48–54 Hz in the 50-Hz condition (*P* < 0.004) (Fig. 2). In the 20-Hz condition, a significant increase of the amplitude was observed around 40 Hz (38–44 Hz, *P* < 0.003), but not around the stimulus frequency. On the other hand, a significant reduction of the amplitude was observed at 28–32 Hz (*P* = 0.01–0.039) in the 20-Hz condition, and at 26–30 Hz (*P* = 0.007–0.035) in the 40-Hz condition (Table 1). As for the hemispheric difference, the overall amplitude was significantly greater for the left hemisphere for all conditions at broad frequencies except the stimulus frequency; for example, at 10–40 and 54–60 Hz for the 50-Hz condition. This was largely due to the greater baseline amplitude in the left hemisphere. Meanwhile, the degree of facilitation at the stimulus frequency of 200–700 ms was greater in the right hemisphere (Fig. 2), which resulted in a small hemispheric difference in the overall amplitude around the stimulus frequencies. A significant hemisphere × PrePost interaction was observed at 32 (*P* = 0.009) and 34 (*P* = 0.004) Hz under the 40-Hz condition; as compared to the pre-stimulus baseline, the amplitude for 200–700 ms was significantly smaller in the left hemisphere at 32 Hz, and significantly greater in the right hemisphere at 34 Hz, which suggested that a reduction at around 30 Hz was predominant in the left hemisphere.

**Table 1:** The ratio of the amplitude relative to the baseline

Condition		26 Hz	28 Hz	30 Hz	32 Hz
20-Hz sound	Lt	0.992 (0.030)	0.988 (0.038)	0.985 (0.046)	0.986 (0.049)
	Rt	0.985 (0.045)	0.980 (0.041)	0.976 (0.041)	0.975 (0.038)
40-Hz sound	Lt	0.986 (0.030)	0.985 (0.027)	0.984 (0.031)	0.985 (0.033)
	Rt	0.986 (0.033)	0.994 (0.031)	0.998 (0.040)	1.009 (0.047)

Data are shown as mean (SD) values

**Figure 3:**
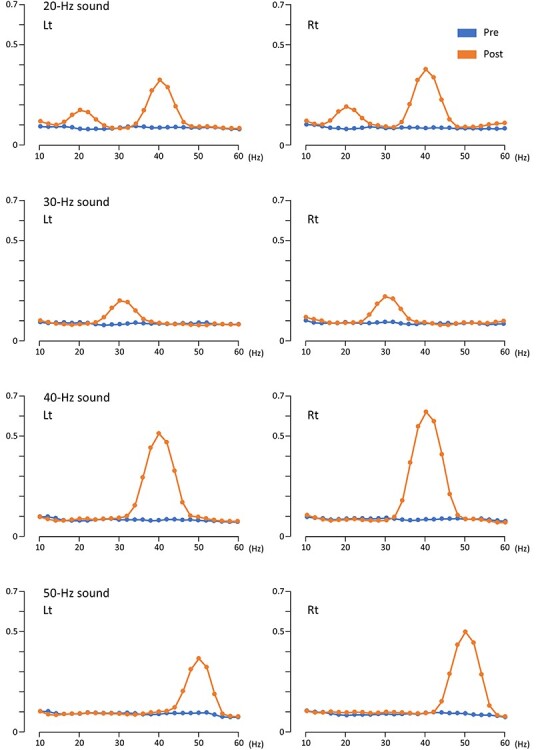
**Changes in the inter-trial coherence of oscillations in the two-dipole model.** The average coherence of the baseline from 500 to 0 ms before the onset of auditory stimulation (Pre) and the average coherence of 200–700 ms (Post) in the left and right hemispheres under each condition are presented here.

Regarding the inter-trial coherence, an increase in coherence with a clear peak was observed in each stimulus frequency (Fig. 3). The average coherence of 200–700 ms at the peak frequencies in four sound conditions were compared using two-way ANOVA with hemisphere and stimulus frequency as independent variables. The results showed a significant difference in hemisphere (F_1,20_ = 28.6, *P* = 6.05 × 10^−6^), stimulus frequency (F_3,18_ = 69.1, *P* = 4.50 × 10^−10^), and interaction effects (F_3,18_ = 14.5, *P* = 4.77 × 10^−5^). Inter-trial coherence at the stimulus frequency increased in the order of 20-, 30-, 50-, and 40-Hz conditions. Post hoc tests showed a significant difference between stimulus frequencies in both hemispheres (*P* < 0.002) except between the 20- and 30-Hz conditions (*P* > 0.99), and right hemispheric dominance in the 40- and 50-Hz conditions (*P* < 0.001), similar to the results of the amplitude of oscillations. In the 20-Hz condition, coherence had clear peaks at 20 and 40 Hz. When two peaks were compared using ANOVA, the coherence was significantly greater for 40 than 20 Hz (F_1,20_ = 40.9, *P* = 3.07 × 10^−6^). The coherence in the right hemisphere was significantly greater (F_1,20_ = 8.20, *P* = 0.01) and there was no interaction (F_1,20_ = 2.98, *P* = 0.10). Next, we examined whether there was a suppression of coherence at 28 and 30 Hz, which were both significant under 20- and 40-Hz conditions in the analysis of the amplitude of oscillations. The average coherence of 200–700 ms in three sound conditions except the 30-Hz condition were compared using two-way ANOVA with hemisphere and stimulus frequency as independent variables for 28 and 30 Hz. The results showed that coherence was not affected by hemisphere (*P* > 0.49), stimulus frequency (*P* > 0.66), and interaction effects (*P* > 0.25) at either 28 or 30 Hz. As shown in Figure 3, owing to the considerably small coherence for the background activity, it appeared to be difficult to observe differences among conditions. Therefore, further analyses were not performed.

### Results of the Multi-Dipole Model Analysis

**Figure 4:**
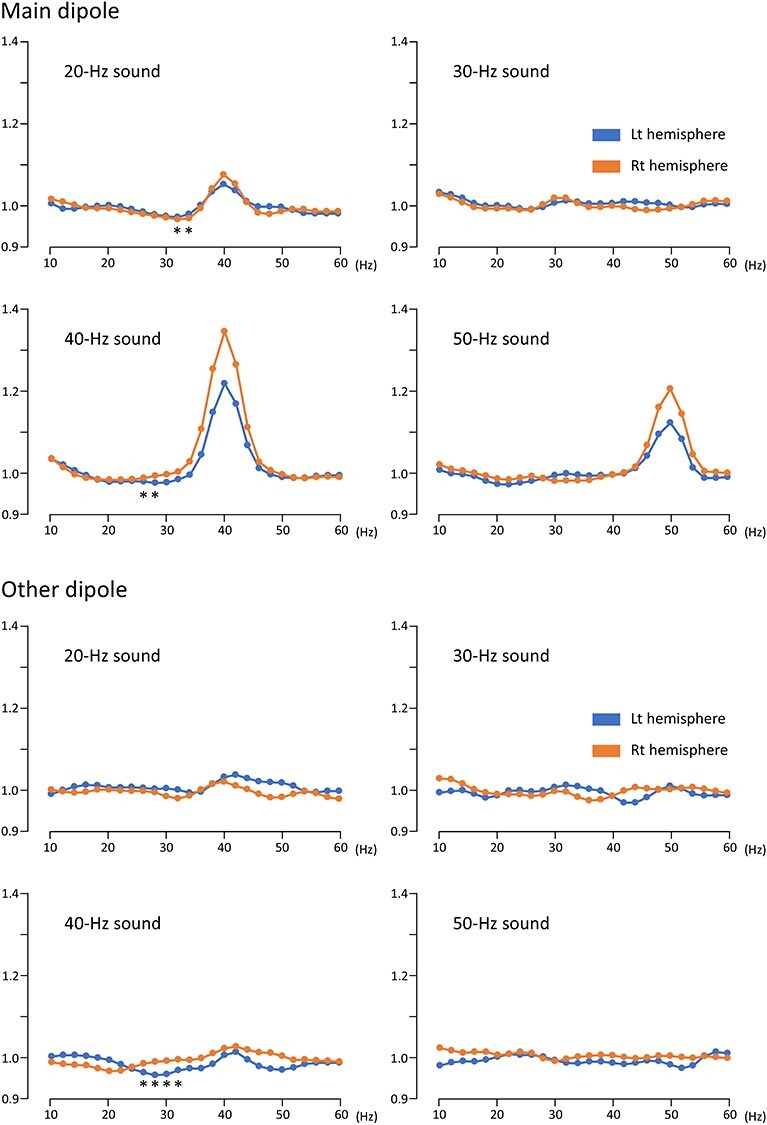
**Changes in the amplitude of oscillations in the multi-dipole model.** The vertical axis indicates the ratio of the amplitude relative to the baseline. Asterisks indicate a significant decrease in the oscillation amplitude (*P* < 0.05).

The results of the two-dipole model analysis showed an increase in the oscillation amplitude at the stimulus frequency and a concomitant decrease at around 30 Hz, suggesting that these two responses were from different groups of neurons. Therefore, explaining the 30 and 40 Hz activities using the same dipole would not be appropriate and we tried to separate them using a multi-dipole analysis. The main dipole was responsible for the enhanced oscillation that occurred at the stimulus frequency, whereas the other dipoles were responsible for the reduction at around 30 in the 40-Hz condition (Fig. 4). As for the other dipoles in the 40-Hz condition, results of paired *t*-test showed a significant decrease in amplitude at 26–32 Hz (*P* = 8.20 × 10^−5^–3.50 × 10^−3^, uncorrected for multiple comparisons) for the left hemisphere. Under the 20-, 30-, and 50-Hz conditions, there was no significant decrease in either hemisphere (*P* > 0.059).

Regarding the main dipole, two-way ANOVAs showed that PrePost significantly affected the oscillation amplitude at 38–44 Hz under the 20-Hz condition, at 36–46 Hz under the 40-Hz condition, and at 44–54 Hz under the 50-Hz condition, which was similar to the results of the two-dipole model. However, a significant reduction was also observed at around 30 Hz under the 20 and 40-Hz conditions; at 32 Hz (*P* = 0.033) and 34 Hz (*P* = 0.032) under the 20-Hz condition, and at 26 Hz (*P* = 0.039) and 28 Hz (*P* = 0.036) under the 40-Hz condition, which indicated that the activities of different groups of neurons between the main dipole and the other dipoles were not completely separate. A significant interaction was found at 34 Hz for the 40-Hz condition (*P* = 0.013); the sound stimulus significantly decreased the oscillation amplitude only in the left hemisphere.

### Results of the all-Sensor Analysis

Thus far, the results of the two analyses suggested that a proportion of the oscillatory activities was suppressed during the 40-Hz sound stimulus. At least some of the activities could be attributed to dipoles around the main dipole responsible for 40-Hz ASSR; however, there remained a possibility that these activities were distributed over widespread brain regions and were not fully reflected in the dipole model. Therefore, we performed an all-sensor analysis to see the distribution of the 40-Hz ASSR and suppression of oscillations at around 30 Hz in more detail.

**Figure 5:**
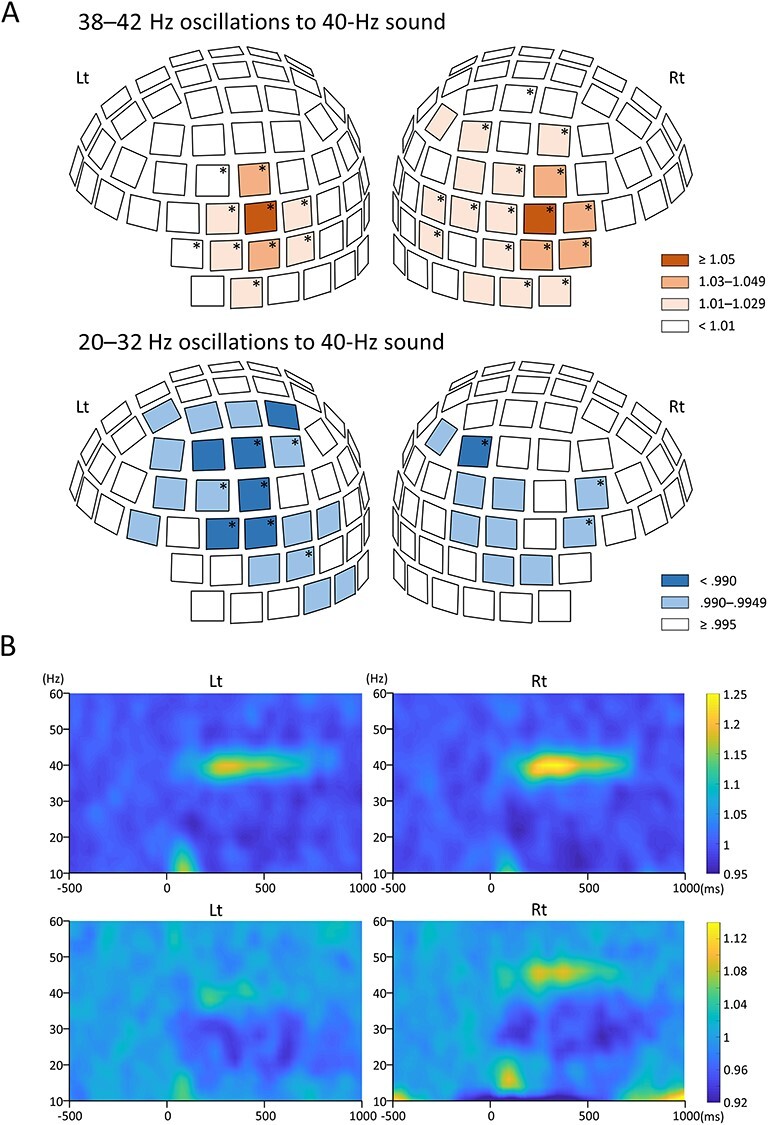
**Results of the all-sensor analysis.** (A) Three-dimensional sensor maps showing the ratio of the oscillation amplitude to the baseline at 38–42 Hz (upper panel) and 20–32 Hz (lower panel) under the 40-Hz sound condition. The sensor locations are aligned across subjects based on the sensor location with the largest 40-Hz ASSR per hemisphere indicated by darkest orange. Asterisks indicate sensors with a significant increase or decrease (*P* < 0.05). Nine sensors (*P* = 4.08 × 10^−7^–0.036, uncorrected for multiple comparisons) in the left hemisphere and 16 sensors (*P* = 1.01 × 10^−7^–0.034) in the right hemisphere that significantly increased the amplitude around 40 Hz. There were seven sensors (*P* = 7.74 × 10^−3^–0.047) in the left hemisphere and three sensors (*P* = 0.011–0.038) in the right hemisphere that significantly decreased the amplitude at 20–32 Hz. (B) The grand-averaged time-frequency maps for the representative sensors show enhanced oscillations at 38–42 Hz (upper panel) and suppression at 20–32 Hz (lower panel) under the 40-Hz sound condition. The sensors with the highest ratio of the amplitude of 200–700 ms to the baseline at 38–42 Hz and with the lowest ratio at the 20–32 Hz amplitude were selected, respectively. The representative sensors of suppression at 20–32 Hz for all subjects were within 25 sensors around the largest oscillations at 38–42 Hz.

Figure 5A shows the mean ratio of oscillatory amplitudes at 200–700 ms relative to the baseline at 20–32 and 38–42 Hz under the 40-Hz condition for sensor elements whose relative positions were corrected across subjects. The center of the 40-Hz ASSR surrounded the Sylvian fissure, and the degree of enhancement was predominant in the right hemisphere. The area of enhancement of the 40-Hz oscillation tended to spread toward the lower temporal area. On the other hand, the area of suppression of 20–32 Hz oscillations surrounded the center of the 40-Hz oscillation and tended to extend to the vertex. Unlike the 40-Hz oscillation, the 20–32 Hz suppression was predominant in the left hemisphere. The distributions of statistically significant changes are indicated by asterisks in Figure 5A, while the time-frequency map averaged across the representative sensors with increased oscillation at 40 Hz and suppressed oscillations at 20–32 Hz is shown in Figure 5B.

## Discussion

This study examined the interactions of gamma oscillations at different frequency bands in humans by using a simple paradigm to induce ASSR. At first, the two-dipole analysis confirmed suppression of low-frequency gamma oscillations at around 30 Hz during the 40-Hz ASSR, which suggested that both oscillations could be explained by a dipole in and near the auditory cortex. However, such a behavior indicated that these two oscillations originate from different groups of neurons. Therefore, as the next step, we attempted to separate them using the multi-dipole analysis. The results supported the expectation by showing that dipoles to explain oscillations other than the main ASSR were estimated to be located around the main dipole of ASSR or sometimes outside the auditory cortex having dipole orientation significantly different from that for the main dipole (*P* < 0.01, discriminant analysis, Figure 1Ab). The results lead us to perform the all-sensor analysis because above results did not fully disclose the distribution of suppression of low-gamma oscillations. We observed that the area of suppression surrounded the main ASSR brain region but the distribution pattern for the two oscillations was slightly different.

Recent studies indicated that different types of interneurons promote distinct rhythms in the neocortex (Cardin 2018). Moreover, it was speculated that reciprocal interactions between somatostatin interneurons and pyramidal neurons generate beta or low-gamma oscillations, whereas interactions between parvalbumin interneurons and pyramidal neurons generate gamma oscillations at faster frequencies; the simultaneous interactions of these two circuits might enable flexible timing control in low or high frequency bands as demand changes (Beierlein et al. 2000; Deans et al. 2001; Chen et al. 2017). As for the 30 and 40-Hz oscillations in question, Middleton et al. (2008) demonstrated with rat slice preparations that two different interneurons have a specific role in the generation of oscillations at these frequencies. It is of note that the present results showed presence of two distinct circuits oscillating at 30 and 40 Hz, and the latter inhibits the former.

The 40-Hz ASSR was elicited not only by the 40-Hz sound but also by the 20-Hz sound, and oscillations around 30 Hz were suppressed by both sounds. These findings indicate that suppression of low-gamma oscillations occurred only when the 40-Hz oscillation was driven. Therefore, activation of the 40-Hz oscillation circuit is presumed to affect the circuit of low-gamma oscillations. Inhibitory interneurons mostly innervate several nearby pyramidal neurons densely and without specificity, often overlapping pyramidal neurons in a “blanket of inhibition” (Fino et al. 2013; Karnani et al. 2014). In the present study, suppression of low-gamma oscillations was observed around the region of maximum amplitude of the 40-Hz oscillation. These results seemed to suggest that the “blanket of inhibition” might contribute to unspecific innervation of pyramidal neurons during the circuit of low-gamma oscillations emitted by inhibitory interneurons, which were driven by the circuit of 40-Hz oscillation. However, 1) distributions of the increased 40-Hz oscillation and decreased low-frequency gamma oscillations differed slightly, 2) the suppression of gamma oscillations was limited to frequencies around 30 Hz, and 3) the 40-Hz oscillation was greater in the right hemisphere, yet suppression of gamma oscillations was greater in the left hemisphere, which indicated that the axons of interneurons in the 40-Hz circuit might induce specific innervation of the nearby low-gamma circuit. It has been determined that some inhibitory interneurons are projected to a remote brain area (Alonso and Köhler 1982) to reach a specific target. The present study found that suppression extended to the parietal cortex while the 40-Hz ASSR originated from the auditory cortex, so it was speculated that suppression was caused by interneurons with a long axon.

The suppression of low-frequency gamma oscillations by activation of 40-Hz oscillation observed in this study may correspond to the phenomenon of specific interaction between oscillations of distinct frequencies, the so-called cross-frequency coupling (Hyafil et al. 2015). There are four kinds of cross-frequency coupling including phase–frequency coupling, phase–phase coupling, phase–amplitude coupling, and amplitude–amplitude coupling (Jensen and Colgin 2007). Among them, our present finding may correspond to amplitude–amplitude coupling. However, the present finding is different from the basic concept of cross-frequency coupling where the slow oscillations affect the fast oscillations. Although cross-frequency coupling may play a functional role in neuronal computation, learning, and communication (Canolty and Knight 2010), little is known about the physiological significance of the interaction of oscillations between different frequencies, including cross-frequency coupling (Hyafil et al. 2015).

There is no room for doubt as to essential contribution of inhibitory interneurons in generating gamma oscillations (Cobb et al. 1995; Whittington et al. 1995; Wang and Buzsáki 1996; Cardin et al. 2009; Sohal et al. 2009; Buzsáki and Wang 2012). In addition, N-methyl-d-aspartic acid (NMDA) receptors are also thought to play a role for gamma oscillations (Grunze et al. 1996; Kinney et al. 2006; Behrens et al. 2007; Homayoun and Moghaddam 2007; Zhang et al. 2008; Belforte et al. 2010). It is particularly important here because Middleton et al. (2008) demonstrated in rats that activation of basket cells via NMDA receptors produced 40-Hz oscillations on one hand and inhibited another type of interneuron responsible for the circuit producing 30-Hz oscillations on the other. Thus, the suppression of low-frequency gamma oscillations by activation of 40-Hz oscillation observed in this study may be modulated via NMDA receptors, as in the study by Middleton et al. (2008).

In the present study, there was a significant increase of the amplitude of oscillation at approximately 40 Hz, but not at 20 Hz in the 20-Hz condition (Fig. 2). The inter-trial coherence increased at 20 Hz but was even greater at 40 Hz (Fig. 3). These findings suggest that there is a specific circuit in the auditory cortex that is activated by 40-Hz rhythm. A previous study examining ASSR to amplitude-modulated tones has shown that at modulation frequencies between 10 and 20 Hz, amplitude of oscillations around 40 Hz were dominant over ASSR at stimulus frequencies (Ross et al. 2000), which supports this idea. In this study, we used a pure tone, which might also be one of the reasons for the lack of significant 20-Hz ASSR. The click tone tends to elicit a greater ASSR than the pure tone, and the amplitude of 20-Hz ASSR is clearly observed in a study using the click tone (Pastor et al. 2002). In fact, the small amplitude of 20-Hz ASSR was observed in this study, although it was not significant (Fig. 2).

There are some limitations in the present study. The first is age-related differences in ASSR. Although the amplitude of ASSR is thought to be affected by age (Rojas et al. 2006), the present sample size was too small to examine it in detail. For example, in the 40-Hz sound condition of the two-dipole model, when subjects were divided into the older (10 subjects, mean 40.6 years) and younger (11 subjects, mean 26.8 years) groups, the amplitude of the 40-Hz oscillation was indeed slightly greater in the younger group, but the difference was not significant (*P* = 0.44). Similarly, suppression of the 30-Hz oscillation tended to be greater for the younger group, but the difference did not reach a significant level (*P* = 0.88). Further studies with larger sample sizes are needed to clarify the effect of age. The second limitation concerns the interhemispheric differences in the suppression of low-gamma oscillations. In the present study, the amplitude of ASSR showed the right hemisphere dominance as already shown (Ross et al. 2005a). In addition, the left hemisphere dominance of the low-gamma suppression and left hemisphere dominance of baseline oscillations in a wide range were found. However, interactions among these hemispheric lateralities are unclear; they might be just caused by the difference in the 40-Hz oscillation between hemispheres. One hypothesis is that properties of NMDA receptors may differ between hemispheres. It is known that asymmetry in the synaptic distribution of NMDA receptor subunits provides the molecular basis for structural and functional asymmetries in the brain (Kawakami et al. 2003; Kawahara et al. 2013). However, further studies using pharmacological and other biological techniques are needed to provide evidence that activation of NMDA receptors is necessary for the interaction between such oscillatory circuits.

## Conclusions

Using a simple paradigm to induce ASSR, the suppression of low-frequency gamma oscillations caused by the activation of 40-Hz oscillations was demonstrated. To the best of our knowledge, the present study is the first to report interactions among gamma oscillations occurring at different frequency bands in humans. Two patterns of gamma oscillations are generated by two different neural circuits, and the 40-Hz circuit might have specific inhibitory innervation to the low gamma circuit. NMDA receptors may be involved in such interactions between gamma oscillations.

## Author Contributions

S.S. and K.I. designed the work. S.S., T.T, T.K., N.T., M.N., and K.I. performed the experiments. S.S. and K.I. analyzed the data. S.S. and K.I. drafted the manuscript. K.O. and T.S. provided valuable critical input on the manuscript. All authors read and approved the manuscript.

## Notes

The authors are grateful to Mr Yasuyuki Takeshima for his technical support. *Conflict of Interest*: None declared.

## Data availability

Raw data were stored at the National Institute for Physiological Sciences, Okazaki, Japan. Derived data supporting the findings of this study are available from the corresponding author on request.

## Financial Support

This study was supported by JSPS KAKENHI Grant Number JP20K16624 to S.S. and JP18K07619 to K.I. and the Cooperative Study Program (2019–16) of the National Institute for Physiological Sciences.
